# Life of the Late Sir Andrew Clark

**Published:** 1900-04-14

**Authors:** Malcolm MacColl

**Affiliations:** Members' Mansions, Victoria Street, S. W.


### Editor's Letter-Box.

[Our Correspondents are reminded that prolixity is a great bar to publication, and that brevity of style and conciseness of statement greatly facilitate early insertion.]

### LIFE OF THE LATE SIR ANDREW CLARK.

Sir,—Will you kindly allow me to send the following brief explanation in reply to "Alpha's" letter in your issue of March 24th ? I undertook the task of writing the life of Sir Andrew Clark, in conjunction with Dr. Allchin, who took charge of the medical side of the great physician's career. After I had written a good part of my share, further research among Sir Andrew's papers and books disclosed so much fresh material that I threw aside all that I had previously written and began afresh. After making satisfactory progress I was stricken down with a serious illness and ordered abroad, and forbidden any work requiring intellectual effort. I am now, thank God, able to resume work, and I hope the life will be ready for the next publishing season. Most of it, in fact, is written, but will require re-writing in parts in order to bring it within the compass of one volume. I can assure "Alpha" that there will be no "burking" in the book, which, I think, will be found full of interest.—I remain, &c.,

Malcolm MacColl.

Members' Mansions, Victoria Street, S.W., April 2nd.

